# EuroQol (EQ-5D-5L) Validity in Assessing the Quality of Life in Adults With Asthma: Cross-Sectional Study

**DOI:** 10.2196/10178

**Published:** 2019-01-23

**Authors:** Gimena Hernandez, Olatz Garin, Alexandra L Dima, Angels Pont, Marc Martí Pastor, Jordi Alonso, Eric Van Ganse, Laurent Laforest, Marijn de Bruin, Karina Mayoral, Vicky Serra-Sutton, Montse Ferrer, ASTRO-LAB Group

**Affiliations:** 1 Health Services Research Group Hospital del Mar Medical Research Institute Barcelona Spain; 2 Department of Paediatrics, Obstetrics and Gynaecology and Preventive Medicine Universitat Autònoma de Barcelona Barcelona Spain; 3 Centro de Investigación Biomédica en Red de Epidemiología y Salud Pública Madrid Spain; 4 Experimental and Health Sciences Pompeu Fabra University Barcelona Spain; 5 Health Services and Performance Research Université Claude Bernard Lyon 1 Lyon France; 6 Pharmaco-Epidemiology Lyon Lyon France; 7 Institute of Applied Health Sciences University of Aberdeen Scotland United Kingdom; 8 Agència de Qualitat i Avaluació Sanitàries de Catalunya Barcelona Spain

**Keywords:** asthma, EQ-5D-5L, EuroQol, health-related quality of life, Web-based survey, validity, patient-reported outcome measures

## Abstract

**Background:**

The EuroQol-5 Dimension (EQ-5D), developed in 1990, is a most widely used generic tool to measure the health-related quality of life (HRQoL) and considered suitable for patients with asthma. In 2009, the EuroQol Group developed a new EQ-5D version to overcome limitations related to its consistently reported high ceiling effect. To enhance the sensitivity for assessing the HRQoL in further patient populations, the number of responses of EQ-5D was increased from 3 to 5 levels (EQ-5D-5L). Moreover, the availability of well-defined requirements for its Web-based administration allows EQ-5D-5L use to monitor the HRQoL in electronic health (eHealth) programs. No study has evaluated the metric properties of the new EQ-5D-5L in patients with asthma yet.

**Objective:**

This study aims to examine the distribution, construct validity, and reliability of the new EQ-5D-5L questionnaire administered online to adults with asthma.

**Methods:**

We evaluated patients with asthma (age: 18-40 years) from a primary care setting in France and England, who self-completed the EQ-5D-5L questionnaire online. The inclusion criteria were persistent asthma defined as >6 months of prescribed inhaled corticosteroids and long-acting beta-agonists or inhaled corticosteroids alone during the 12 months prior to inclusion. The EQ-5D index was obtained by applying the English preference value set for the new EQ-5D-5L and the French 3L-5L crosswalk value set. Both value sets produced single preference-based indices ranging from 1 (best health state) to negative values (health states valued as worse than death), where 0=death, allowing the calculation of quality-adjusted life years. Responses to dimensions and index distribution, including ceiling and floor effects, were examined. The construct validity was assessed by comparing the means of known groups by analyses of variance and calculation of effect sizes.

**Results:**

Of 312 patients answering the baseline Web-based survey, 290 completed the EQ-5D-5L (93%). The floor effect was null, and the ceiling effect was 26.5% (74/279). The mean EQ-5D-5L index was 0.88 (SD 0.14) with the English value set and 0.83 (SD 0.19) with the French 3L-5L crosswalk value set. In both indices, large effect sizes were observed for known groups defined by the Asthma Control Questionnaire (1.06 and 1.04, *P*<.001). Differences between extreme groups defined by chronic conditions (*P*=.002 and *P*=.003 for the English value set and French 3L-5L crosswalk value set, respectively), short-acting beta-agonists (SABAs) canisters in the last 12 months (*P*=.02 and *P*=.03), or SABA use during the previous 4 weeks (*P*=.03 and *P*=.01) were of moderate magnitude with effect sizes around 0.5.

**Conclusions:**

The new EQ-5D-5L questionnaire has an acceptable ceiling effect, a good construct validity based on the discriminant ability for distinguishing among health-related known groups, and high reliability, supporting its adequacy for assessing the HRQoL in patients with asthma. EQ-5D-5L completion by most Web-based respondents supports the feasibility of this administration form.

## Introduction

The impact of asthma on the patients’ health has been traditionally assessed by either clinical markers or functional tests [1]. Patient-reported outcome measures, such as symptom control or health-related quality of life (HRQoL), have shown to be useful for clinical management, understanding disease impact on the patients’ functional status and well-being, and cost-effectiveness analyses [2]. Hence, international guidelines for asthma diagnosis and treatment have emphasized that treatment goals should include the improvement of the patients’ HRQoL [3].

In asthma, disease-specific HRQoL measures have been more widely used than generic ones, as they could be more sensitive. Adding generic HRQoL domains important to patients with asthma has been proposed [4] because asthma-specific HRQoL instruments measure similar contents to those covered by asthma control questionnaires [5,6] such as symptoms and activity limitations. Generic HRQoL instruments are broad measures that can be applied in patients with various conditions and the general population. The EuroQol-5 Dimensions (EQ-5D), developed in 1990 by the EuroQol Group, is one of the most widely used generic tools owing to its low respondent burden, good psychometric properties, and econometric development [7-9]. In addition, the availability of well-defined requirements for its Web-based administration by multiple devices, such as personal computer, tablet, or smartphones, makes this instrument adequate for monitoring the HRQoL in eHealth programs [10].

The EQ-5D was considered a suitable generic measure in a systematic review [11] of patient-reported outcome measures for patients with asthma. This health status measure allows the calculation of quality-adjusted life years (QALYs) when society preferences are applied and cost-utility analysis in economic evaluations [12-14]. However, to the best of our knowledge, only 3 studies have evaluated its psychometric properties in patients with asthma [15-17]. Garratt et al [16] showed a moderate EQ-5D association with asthma-specific HRQoL instruments and external variables such as smoking status and education level. Oga et al [15] and McTaggart-Cowan et al [17] reported a high ceiling effect (59% and 50% of the sample with the maximum score, respectively) questioning the usefulness of the EQ-5D in asthmatic patients. In fact, limitations related to the high ceiling effect have also been consistently reported for the EQ-5D in other chronic conditions such as chronic obstructive pulmonary disease [18], osteoarthritis [18], diabetes [19], and coronary heart disease [20].

The traditional EQ-5D descriptive system, composed of 5 dimensions with 3 levels of severity, defines 243 distinct health states resulting from all the possible combinations (ie, 3^5^); this is a low number compared with other generic preference-based instruments such as the Health Utilities Index [21] or the SF-6D [22] with 972,000 and 18,000 possible combinations, respectively. To improve its sensitivity, the EuroQol Group developed a new EQ-5D version, by increasing the number of responses from 3 to 5 levels, known as EQ-5D-5L, with 3125 health states (ie, 5^5^) [23].

The new EQ-5D-5L has already been tested in some disease-specific samples, such as patients with cancer [24,25] and with hepatitis [26], showing a better discrimination capability and lower ceiling effects than the traditional 3-level version (11% vs 17% [24], 9.7% vs 16.8% [25], and 21.6% vs 38.3% [26]). However, to date, no study has evaluated metric properties of the new 5-level EQ-5D in patients with asthma. Hence, this study aims to examine the distribution, construct validity, and reliability of the new EQ-5D-5L administered online to adults with asthma.

## Methods

### Setting and Study Population

In this study, we analyzed baseline data of adults (age: 18-40 years) enrolled in the ASTRO-LAB cohort who completed the EQ-5D-5L questionnaire. The ASTRO-LAB project is a prospective longitudinal study of asthmatic patients designed to provide new evidence regarding the safety of long-acting beta-agonists (LABAs) in routine primary care in France and the United Kingdom. Details of the study are described elsewhere [27].

The inclusion criteria were as follows: persistent asthma and age <40 years. Patients were considered to have persistent asthma when they had >6 months of prescribed treatment with inhaled corticosteroids (ICs) and LABAs or ICs alone during the 12 months prior to inclusion. Persistent asthma requires controller therapy on a regular basis, whereas intermittent asthma can be treated with rescue medication as needed. The ASTRO-LAB persistent asthma definition was based on a minimal prescription duration level of antiasthmatic drugs because this method is considered less biased than the practitioner’s classification of asthma, and it is frequently used in database studies [28]. The ASTRO-LAB project’s age limit was chosen to minimize the recruitment of patients with other comorbid conditions frequent at older ages, most importantly chronic obstructive pulmonary disease, often overlapped and difficult to exclude without specific tests.

The exclusion criteria were as follows: chronic oral corticosteroid use (≥15 consecutive days 3 months before inclusion), history of omalizumab therapy, and any other concomitant chronic respiratory disease (chronic obstructive pulmonary disease, cystic fibrosis, pulmonary fibrosis, bronchiectasis, or tuberculosis). Owing to ASTRO-LAB’s main focus being LABAs safety, the abovementioned criteria based on the administration of other medications aimed at avoiding confounding with their adverse effects, implying that most patients with severe persistent asthma were excluded.

The ASTRO-LAB study has been approved by the Ethics and Regulatory Boards in France and the United Kingdom and was conducted in accordance with the Declaration of the World Medical Association. In France, approval was obtained from CCTIRS (Comité consultatif sur le traitement de l’information en matière de recherche dans le domaine dela santé) on November 21, 2012 (Dossier N°12702), and the authorization from Commission Nationale d’Informatique et Liberté was obtained in May 17, 2013 (DR-2013-264). In the United Kingdom, according to the UK Research Governance Framework, the study was submitted to The West London Research Ethics Committee (REC), and the final approval was obtained on April 15, 2013 (REC Reference 12/LO/20139). Following the UK regulatory process, the ASTRO-LAB consortium submitted the protocol to the National Institute for Health Research Clinical Research Network (NIHR CRN) to launch the review by Primary Care Trust local sites. The first local approval was granted by the West London Primary Care Consortium on May 22, 2013. Informed consent was obtained from all participants prior to inclusion.

### Measurement Instruments

Clinical data were extracted from medical records, and patient-reported information was obtained by the following 2 administration modes: patient-completed Web-based survey and telephone interviews with patients performed by trained interviewers. The EQ-5D-5L was only administered in the Web-based survey.

#### Clinical Data

Information on age, gender, body mass index, comorbidity, and treatment prescribed was obtained; in France, general practitioners completed a Web-based survey at patient inclusion, while in the United Kingdom, this information was directly extracted from medical records. The history of 4 associated pathologies (allergic rhinitis, nasal polyps, anxiety or depression, and gastroesophageal reflux), was registered and transformed into a count variable. The total number of short-acting beta-agonist (SABA) canisters prescribed in the 12 months prior to inclusion was transformed into a variable of 3 categories—0, 1-4, and ≥5 canisters.

#### Patient-Completed Web-Based Survey

Patients received instructions during the recruitment contact to self-complete a Web-based survey, which included the EQ-5D-5L to measure the HRQoL and sociodemographic data, such as their highest level of education and current work situation, among others.

The EQ-5D-5L is a brief, multiattribute, generic, health status measure composed of 5 questions with Likert response options (descriptive system) and a visual analog scale (EQ-VAS). The latter asks patients to rate their own health from 0 to 100 (the worst and best imaginable health, respectively). The descriptive system covers 5 dimensions of health (mobility, self-care, usual activities, pain or discomfort, and anxiety or depression) with 5 levels of severity in each dimension (no problems, slight problems, moderate problems, severe problems, and unable to perform or extreme problems).

Preference value sets used to obtain the index of the EQ-5D-5L were the 3L-5L crosswalk from the French 3L version [29], and the new EQ-5D-5L value set from England [30]. In both cases, single preference-based indices were produced ranging from 1 (the best health state) to negative values (health states valued as worse than death), where 0=death. The minimal important difference for the EQ-5D index was estimated as 0.07 [31].

#### Telephone Interviews

The telephonic interviews were computer-assisted to standardize the process. Trained interviewers administered questions to patients about their asthma control and treatment use, among others. Asthma control is defined as the extent to which the manifestations of asthma can be observed in a patient, or have been reduced or removed by treatment [32,33]; it reflects the suitability of the asthma treatment.

The Asthma Control Questionnaire (ACQ) is composed of 7 items—the top scoring 5 symptoms, FEV_1_% predicted, and daily rescue bronchodilator use. A shorter version called ACQ-symptoms only [34] was developed to use when it is not feasible to collect data about the last 2 items, as in ASTRO-LAB. It assesses the frequency of the 5 asthma symptoms during the previous week on a 7-point Likert scale (0=no impairment, 6=maximum impairment). The overall score, calculated as the mean of item responses, ranges from 0 to 6. A score <0.75 was defined as well-controlled asthma; 0.75-1.5 as intermediate control; and >1.5 as not well-controlled asthma [35]. The results generated by the short versions have shown to be very similar to those of the complete ACQ, as well as its measurement properties (reliability, responsiveness, internal consistency, construct validity, and interpretability) [34].

The following question was asked to patients with SABA therapy prescription: “How often have you usually taken your ‘reliever medication’ (brand name) in the past 4 weeks? Every day; almost every day; once or twice every week; less than once a week; or I don’t know.”

### Analytic Strategy

Sample characteristics were described by calculating percentages, or mean (SD) values, according to the variable type (detailed in tables and figures). To examine the nonresponse bias, subjects who completed the Web-based survey were compared with those who had not completed this survey by a t test and chi-square test.

We calculated the percentages of responses to each EQ-5D-5L dimension. To examine the distribution of the EQ-5D index, we calculated statistics of central tendency, dispersion, asymmetry, and tail extremity, as well as the proportion and 95% CI of the individuals in the best possible (ceiling) and the worst possible (floor) health states [36]. To assess the reliability based on the internal consistency, the Cronbach alpha coefficient was estimated.

The construct validity examines whether the instrument adequately assesses the concept that it intends to measure [37], in this case the HRQoL. The strategy to evaluate the construct validity based on known groups consists of testing the ability of the instrument to discriminate among groups previously hypothesized as differing in the concept measured. The following variables were chosen to test the instrument’s capacity to discriminate, as differences have been consistently shown in the HRQoL among groups defined by them [1,15,38,39]—the number of chronic conditions (as an indicator of general health), number of SABA canisters prescribed in the previous year, frequency of SABA inhaler use during the previous 4 weeks, and ACQ scores (as 3 indicators of asthma control). We hypothesized that asthma patients with worse general health or less asthma control report worse HRQoL.

To evaluate the discriminative capacity of the EQ-5D index and EQ-VAS among the known groups mentioned above, mean scores were compared using one-way analysis of variance and the Tukey studentized range (honestly significant difference) test for post-hoc comparisons; alpha was set at.05. To assess the magnitude of the differences Cohen effect sizes were calculated. General guidelines define an effect size of 0.2 as small, 0.5 as moderate, and 0.8 as large [40]. All analyses were conducted using the statistical package SPSS (IBM SPSS Statistics for Windows, Version 23.0, IBM Corp)

## Results

### Study Sample

Of 581 subjects with asthma aged 18-40 years composing the ASTRO-LAB cohort, 312 filled in the baseline Web-based survey (Web-based participation rate, 53.7%), but 22 of these did not complete the EQ-5D-5L (questionnaire nonresponse rate, 7.0%). Of 290 who fulfilled the EQ-5D-5L, 11 were excluded because they had missing data on all the variables selected to define known groups; hence, 279 patients were finally included in this analysis. Table 1 shows patients’ baseline characteristics, comparing the included subjects with excluded ones (mainly because of not responding to the Web-based survey). Most of the included subjects were from France and had been treated with fixed-dose combinations of LABA and IC. More than half of them had completed a bachelor degree (66.9%, 184/275), and 72.6% (201/277) were employed in their usual jobs. These 2 variables were only available for patients included in the analysis, as they were recorded in the Web-based questionnaire. Nonrespondents were younger (aged 29.8 vs 31.0 years, *P*=.03), and presented higher ACQ mean scores (worse control) in comparison to respondents but did not differ in body mass index, treatment, number of other chronic conditions, SABA canisters prescribed last year, and frequency of SABA used in the previous 4 weeks.

### 5-Level EuroQoL-5 Dimension Version Distribution

**Figure 1:**
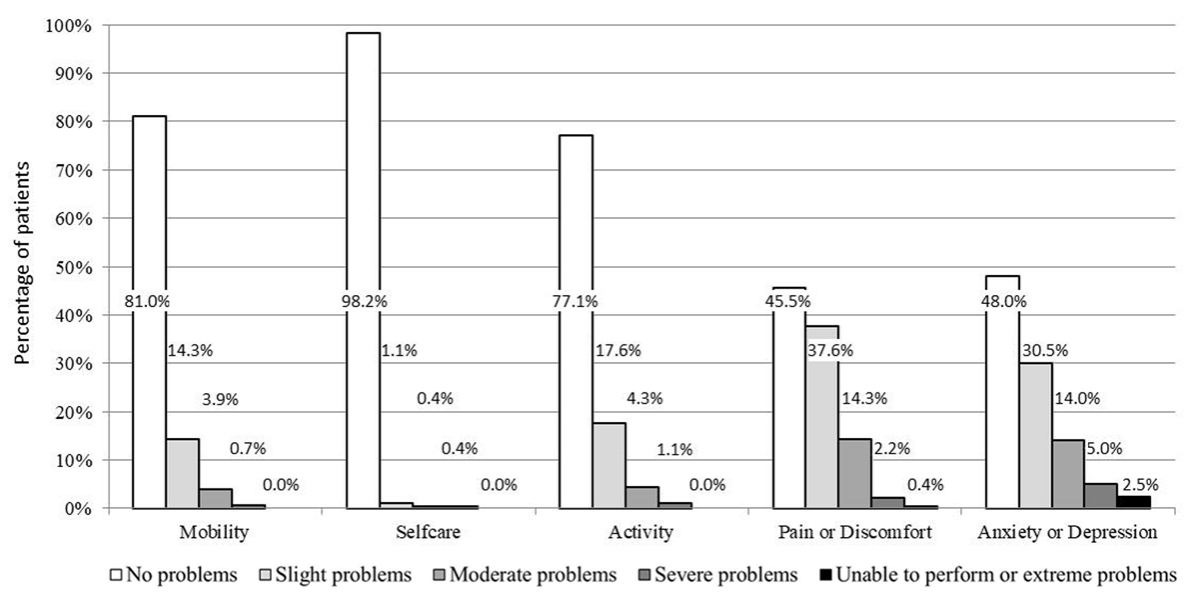
The percentage of patients’ responses to each dimension.

Figure 1 shows the percentages of responses to each EQ-5D-5L dimension. Most subjects reported “no problems” in mobility (81.0%, 226/279) and self-care (98.2%, 274/279) dimensions, while only around half of the subjects endorsed this category in pain or discomfort (45.5%, 127/279) and anxiety or depression (48.0%, 134/279) dimensions. The “extreme problems” category was endorsed by 1 subject for pain and 7 for anxiety or depression.

Table 2 shows the distribution characteristics of EQ-5D-5L indices. In our sample, the EQ-5D-5L index constructed with the English value set ranged from 0.16 to 1 and from –0.074 to 1 when constructed with the French 3L-5L crosswalk value set. The mean was 0.88 (SD 0.14) for the English index and 0.83 (SD 0.19) for the French one. The Kurtosis statistics of 5.62 and 3.26, with skewness of –2.06 and –1.63, indicated that the asymmetry to the right part of the distribution and the tail extremity were greater in the index constructed with the English EQ-5D-5L value set. The floor effect was null, and the ceiling effect was 26.5% (74/279). Cronbach alpha coefficient was .69, achieving the recommended standard [36,37].

### 5-level EuroQoL-5 Dimension Version Construct Validity

Table 3 presents results on the construct validity of the EQ-5D-5L based on known groups. Both EQ-5D-5L indices showed statistically significant different means for all known groups evaluated, while EQ-VAS only showed statistically significant differences among groups defined by ACQ scores. The mean EQ-5D-5L index for asthmatic patients decreased significantly with an increase in the number of other chronic conditions from 0.91 to 0.82 with the English value set and from 0.86 to 0.75 with the French 3L-5L crosswalk. The effect size between patients with none and those with ≥2 other chronic conditions were 0.62 and 0.60 (moderate) with EQ-5D-5L indices. In addition, effect sizes were moderate between extreme groups defined by SABA canisters prescribed in the previous year (0.58 and 0.46), and by the SABA frequency during the last 4 weeks (both 0.5). Finally, among groups defined by ACQ scores, the effect size between well-controlled and intermediately controlled asthma was moderate (0.44 and 0.47) and large between well- and not well-controlled asthma (1.06 and 1.04).

**Table 1:** The characteristics of included and excluded subjects.

Characteristics		Included patients (n=279)	Excluded patients (n=302)	*P* value	
**Age (years), mean (SD)**		31.0 (6.7)	29.8 (6.7)	.03	
	<25, n (%)	62 (22.2)	85 (28.1)	.10	
	25-34, n (%)	119 (42.7)	133 (44.0)		
	≥35, n (%)	98 (35.1)	84 (27.8)		
**Gender, n (%)**					.47
	Male	110 (39.4)	128 (42.4)		
	Female	169 (60.6)	174 (57.6)		
**Country**					.01
	France, n (%)	222 (79.6)	264 (87.7)		
	United Kingdom, n (%)	57 (20.4)	37 (12.3)		
	Missing (n)	0	1		
**Body mass index (kg/m^2^), mean (SD)**		25.2 (6.2)	25.4 (5.8)	.79	
	Missing (n)	127	107		
**Treatment with, n (%)**					.18
	LABA^a^	11 (3.9)	9 (3.0)		
	ICs^b^	71 (25.4)	60 (19.9)		
	LABA+ICs in separate inhalers	37 (13.3)	33 (10.9)		
	Fixed LABA and ICs combination	160 (57.3)	200 (66.2)		
**Other chronic conditions**					.51
	0 conditions, n (%)	66 (41.5)	80 (39.2)		
	1 condition, n (%)	62 (39.0)	91 (44.6)		
	≥2 conditions, n (%)	31 (19.5)	33 (16.2)		
	Missing (n)	120	98		
**Number of SABA^c^ canisters prescribed (last year)**					.75
	0 canisters, n (%)	119 (53.6)	133 (50.2)		
	1-4 canisters, n (%)	78 (35.1)	100 (37.7)		
	≥5 canisters, n (%)	25 (11.3)	32 (12.1)		
	Missing (n)	57	—^d^		
**Frequency of SABA use reported by patient (last 4 weeks)**					.63
	Less than once a week, n (%)	166 (61.9)	171 (65.5)		
	Once or twice every week, n (%)	71 (26.5)	60 (23.0)		
	Almost every day or every day, n (%)	31 (11.6)	30 (11.5)		
	Missing (n)	11	41		
**Asthma Control Questionnaire, mean (SD)**		1.01 (0.92)	1.35 (1.01)	<.001	
	Well-controlled (<0.75), n (%)	119 (44.6)	89 (34.1)	<.001	
	Intermediate (0.75-1.5), n (%)	82 (30.7)	63 (24.1)		
	Not well-controlled (>1.5), n (%)	66 (24.7)	109 (41.8)		
	Missing (n)	12	41		
**Highest education**					Not calculated
	Secondary school or less, n (%)	13 (4.7)	—		
	Sixth form or college, n (%)	41 (14.9)	—		
	Bachelor degree, n (%)	184 (66.9)	—		
	Postgraduate, n (%)	37 (13.5)	—		
	Missing (n)	4	—		
**Work status**					Not calculated
	Employed at usual job, n (%)	201 (72.6)	—		
	On light duty or restricted work, n (%)	1 (0.4)	—		
	Paid leave or sick leave, n (%)	4 (1.4)	—		
	Unemployed because of other reason, n (%)	23 (8.3)	—		
	Student (school, college, university), n (%)	35 (12.6)	—		
	Keeping house or homemaker, n (%)	7 (2.5)	—		
	Retired, n (%)	0 (0.0)	—		
	On disability, n (%)	6 (2.2)	—		
	Missing (n)	2	—		

^a^LABA: long-acting beta-agonist.

^b^IC: inhaled corticosteroid.

^c^SABA: short-acting beta-agonist.

^d^Indicates missing data.

**Table 2:** The distribution of 5-level EuroQoL-5 dimension version indices (n=279). Cronbach alpha coefficient was .69.

Statistics	EQ-5D-5L^a^ (English value set)	EQ-5D-5L (French 3L-5L crosswalk value set)
Theoretical range	–0.28097 to 1	–0.530 to 1
Observed range	0.160 to 1	–0.074 to 1
Mean (SD)	0.88 (0.14)	0.83 (0.19)
Median (interquartile range)	0.92 (0.84 to 1.00)	0.91 (0.71 to 1.00)
Kurtosis (SE)	5.62 (0.29)	3.26 (0.29)
Skewness (SE)	–2.06 (0.15)	–1.63 (0.15)
Floor effect (%)	0	0
Ceiling effect (%)	26.5	26.5

^a^EQ-5D-5L: EuroQol-5 Dimensions-5 Levels.

**Table 3:** The construct validity of 5-level EuroQoL-5 dimension version.

Constructs		EQ-5D-5L^a^ index (English value set)									EQ-5D-5L index (French 3L-5L crosswalk)								EuroQol visual analog scale			
		Mean (SD)	Effect size (95% CI)								Mean (SD)				Effect size (95% CI)				Mean (SD)		Effect size (95% CI)	
**Other chronic conditions**																						
	0 chronic conditions	0.91 (0.11)		Reference			0.86 (0.14)					Reference						78.91 (14.85)			Reference	
	1 chronic condition	0.89 (0.10)		0.14 (–0.21 to 0.48)			0.85 (0.15)					0.05 (–0.26 to 0.36)						79.08 (13.23)			0.06 (–0.25 to 0.37)	
	≥2 chronic conditions	0.82 (0.13)		0.62 (0.18 to 1.06)			0.75 (0.20)					0.60 (0.18 to 1.02)						72.94 (17.22)			0.37 (–0.04 to 0.79)	
	*P* value	.002^b,^^c^		—^d^			.003^b,c^					—						.12			—	
**Number of SABA^e^** **canisters prescribed (last year)**																						
	0 canisters	0.89 (0.11)				Reference			0.85 (0.15)				Reference				78.84 (12.90)					Reference
	1-4 canisters	0.87 (0.14)				0.11 (–0.18 to 0.39)			0.82 (0.19)				0.19 (–0.07 to 0.45)				76.64 (17.93)					0.21 (–0.06 to 0.47)
	5 or more canisters	0.81 (0.17)				0.58 (0.14 to 1.01)			0.76 (0.22)				0.46 (0.05 to 0.86)				72.00 (24.52)					0.47 (0.07 to 0.88)
	*P* value	.02^b^				—			.03^b^				—				.15					—
**Frequency of SABA use reported by patient (last 4 weeks)**																						
	Less than once a week	0.82 (0.19)		Reference				0.74 (0.23)						Reference		71.45 (19.85)				Reference		
	Once or twice a week	0.87 (0.15)		0.17 (–0.11 to 0.44)				0.81 (0.21)						0.29 (0.03 to 0.55)		78.08 (12.92)				0.07 (–0.19 to 0.32)		
	Almost every day or every day	0.89 (0.12)		0.50 (0.11 to 0.89)				0.85 (0.16)						0.50 (0.15 to 0.84)		78.61 (16.26)				0.37 (0.03 to 0.71)		
	*P* value	.03^b^		—				.01^b^						—		.07				—		
**Asthma Control Questionnaire**																						
	Well-controlled (<0.75)	0.93 (0.10)			Reference					0.91 (0.13)				Reference				81.65 (13.80)		Reference		
	Intermediate (0.75-1.5)	0.87 (0.11)			0.44 (0.15 to 0.72)					0.81 (0.15)				0.47 (0.22 to 0.73)				79.18 (11.92)		0.15 (–0.11 to 0.40)		
	Not well-controlled (>1.5)	0.78 (0.19)			1.06 (0.74 to 1.38)					0.69 (0.24)				1.04 (0.75 to 1.32)				68.39 (20.23)		0.79 (0.51 to 1.08)		
	*P* value	<.001^b,c,f^			—					<.001^b,c,f^				—				<.001^b,c^		—		

^a^EuroQol-5 Dimensions-5 Levels.

^b^First category (reference) versus third category.

^c^Second category versus third category.

^d^*P* value not necessary as the CI was calculated.

^e^SABA: short-acting beta-agonist.

^f^First category (reference) versus second category.

## Discussion

### Principal Findings

To the best of our knowledge, this is the first study evaluating metric properties of the new EQ-5D-5L in patients with asthma. In this study, this generic preference-based instrument showed adequate distribution and reliability, with 26.5% (74/279) of patients reporting the best possible health state (ceiling effect). In addition, it showed good construct validity, given its capacity of discriminating among groups differing in the number of chronic conditions and symptom control. The distribution of the EQ-5D-5L index was less skewed than the previously published one for the 3-level version owing to its lower ceiling effect [15,17].

### Comparison of Web-Based Participation Rate With Prior Work

In this study, 53.7% (312/581) of participants completed the Web-based baseline survey, and almost all of these completed the EQ-5D-5L (92.9%, 290/312). The internet era has led to implementing Web-based surveys to take advantage of the known benefits such as completeness [41,42], low expenses [43], and better data management. Nevertheless, there are still some barriers to Web-based self-completion, which could produce low response rates and selection bias. Although the reported participation rate varied a lot across Web-based surveys [41,44,45], the 53.7% in this study is similar to those reported by other studies comparing different modes of data collection, such as 64.2% and 53.3% participation rates reported by Kongsved et al [41] and Hohwu et al [46] studies. Remarkably, both studies showed a slightly better response rate with the paper mode—73.2% versus 64.2% [41] and 56.2% versus 53.3% [46]. In the ASTRO-LAB cohort, the high overall respondent burden (participants were asked to respond to yearly Web-based surveys, 4-monthly telephone interviews, and monthly short message service text messages) could have affected the response rate.

### Comparison With Prior Studies Evaluating the EuroQoL-5 Dimension Version in Patients With Asthma

Despite being higher than the 15% [36] established for the ceiling effect, 26.5% (74/279) of patients with mild-to-moderate persistent asthma in the best possible health state in our sample was considerably lower than that reported in prior studies using the traditional EQ-5D-3L in paper-and-pencil administration [15,17]. A ceiling effect of 59% was described in Japanese patients with mild-to-severe asthma treated with ICs [15], and 50% in Canadian patients with mainly mild-to-moderate self-reported asthma [17]. In addition, our findings showed a lower proportion of patients with no problems in most dimensions than those reported by the 3-level version [15]—81.0% (226/279) versus 90.7% in mobility, 77.1% (215/279) versus 85.2% in activity, 45.5% (127/279) versus 74.1% in pain or discomfort, and 48.0% (134/279) versus 77.8% in anxiety or depression. The other 2 studies on the EQ-5D-3L in asthma [16,17] did not report percentage distributions for each dimension. This lower endorsement of the top response option when compared with results from previous studies with the EQ-5D-3L suggests that the “no problems” category (level 1 out of 3) is partially redistributed to the following intermediate category, “slight” problems (level 2 out of 5), in the new 5-level version. However, head-to-head studies are needed to ensure that the new 5L version’s better properties we have observed, compared with results from previous EQ-5D-3L studies [15,17], are not explained by differences in patients’ characteristics or design issues.

Studies that directly elicit preferences from representative general population samples to derive value sets for the new EQ-5D-5L, using a harmonized protocol, have already been published for several countries [30,47-51], but they are not yet developed in many others, including France. The EuroQol Group developed the 3L-5L crosswalk value sets as a temporary solution to estimate the EQ-5D-5L in such a situation [29]. The difference between both indices in the negative extreme of the theoretical range (–0.28 and –0.53) is explained by the method used for the elicitation of the societal preference values to derive the value set: time trade-off in the French general population for the 3L version [52], and the composite method of time trade-off with discrete choice experiments in the UK general population for the new 5L version [23,30]. Our findings show that the mean EQ-5D-5L indices obtained with both value sets are quite similar (0.88 and 0.83), supporting that the 3L-5L crosswalk is a good interim solution to calculate the EQ-5D-5L index, until definitive EQ-5D-5L value sets are available.

The EQ-5D-5L index could discriminate among different known groups in the hypothesized direction. In all the variables evaluated, differences between extreme groups ranged from 0.07 to 0.2, therefore being equal to or higher than the minimal important difference, previously estimated as 0.07 [31]. The magnitude was moderate for differences among groups defined by the presence of other chronic conditions and SABA use or prescription and large for differences between patients with well- and not well-controlled asthma measured with the ACQ. McTaggart-Cowan et al [17], with the traditional EQ-5D-3L in patients with asthma, also showed differences between extreme groups >0.07, ranging from 0.07 to 0.18. It was not possible to directly compare effect sizes with this study [17], as the variables to define known groups were different. Mc Taggart-Cowan et al reported a correlation of 0.37 for the ACQ with the EQ-5D-3L index [17], similar to the 0.43 found in our study with the EQ-5D-5L index. These findings indicate a good construct validity for the EQ-5D-5L index, which, in general, presented a greater discriminant capacity than the EQ-VAS among the known groups evaluated.

### Limitations and Strengths

Some potential limitations of this study need to be considered. First, a direct comparison with the EQ-5D-3L was not possible. Although previous EQ-5D-3L studies in asthma patients [15-17] showed higher ceiling effects and lower discriminatory properties than ours with the EQ-5D-5L, differences among studies regarding patients’ and design characteristics cannot be discarded. Second, as no asthma-specific HRQoL measure was included in this study, we were unable to compare the generic EQ-5D-5L with them. Studies evaluating the EQ-5D-3L in comparison to the Asthma Quality of Life Questionnaire [15-17] or the Newcastle Asthma Symptoms Questionnaire [16] showed that these disease-specific instruments were more sensitive to change. Further head-to-head studies comparing the EQ-5D-5L with disease-specific instruments are needed, mainly to compare responsiveness. Third, the usability of online versus other methods of survey administration could not be evaluated because all patients completed the Web-based EQ-5D-5L. Fourth, because the ASTRO-LAB project only included patients with mild-to-moderate persistent asthma, the generalizability of our results to those with intermittent or severe persistent asthma is uncertain. The generalizability is also uncertain to patients older than 40 years. Finally, it is important to note that 46.3% (269/581) of participants in the ASTRO-LAB project did not answer the Web-based survey. No differences in sociodemographic characteristics, treatment, and comorbidity were found between respondents and nonrespondents, and differences detected in asthma control were minor. However, there could be differences in other characteristics, which have not been measured such as personality or other psychological traits.

This study has several strengths that need to be highlighted. First, embedding this study in an observational cohort in routine care allowed us to select several appropriate known groups for evaluating the EQ-5D-5L’s construct validity in patients with asthma. The relationship between comorbid chronic conditions and health is well established, and the associations of symptoms control [53] with the HRQoL have been extensively studied in this population. Furthermore, the ACQ, validated in 50 languages, is one of the most widely accepted instruments for measuring asthma control [54].

### Conclusions

In summary, our results provide support to the construct validity of the EQ-5D-5L administered online to patients with asthma, based on its discriminant ability for distinguishing among health-related known groups, as well as its lower ceiling effect than previously reported for the traditional 3-level version [15,17]. The completion of the EQ-5D-5L by most of the Web-based survey respondents supports the feasibility of this administration form. As it was developed as a preference-based health status measure, the EQ-5D-5L index allows combining both length and quality of life, and calculates QALYs to measure health outcomes in economic evaluations. All these findings suggest that the new EQ-5D with 5 levels is a promising instrument to compare the efficiency of different programs or treatment strategies for asthma patients. Nevertheless, further studies are recommended to evaluate the responsiveness over time of the EQ-5D-5L among patients with asthma.

## Abbreviations

ACQ: Asthma Control Questionnaire
EQ-5D-5L: 5-level EuroQoL-5 dimension version
EQ-VAS: EuroQol-visual analog scale
HRQoL: Health-Related Quality of Life
IC: inhaled corticosteroid
LABA: long-acting beta-agonists
SABA: short-acting beta-agonists
